# Targeting the overexpressed ROC1 induces G2 cell cycle arrest and apoptosis in esophageal cancer cells

**DOI:** 10.18632/oncotarget.16250

**Published:** 2017-03-16

**Authors:** Jingyang Zhang, Shuo Li, Zhaoyang Shang, Shan Lin, Peng Gao, Yi Zhang, Shuaiheng Hou, Saijun Mo, Wenbo Cao, Ziming Dong, Tao Hu, Ping Chen

**Affiliations:** ^1^ College of Basic Medical Sciences, Zhengzhou University, Collaborative Innovation Center of Henan Province for Cancer Chemoprevention, Zhengzhou, 450001, China

**Keywords:** ROC1, cell cycle, apoptosis, esophageal cancer, NOXA

## Abstract

Recent reports showed that regulator of Cullins-1 (ROC1) play an important role in tumor progression in a tumor-specific manner. However, the role and mechanism of ROC1 in esophageal cancer remains elusive. Here we demonstrated that ROC1 was overexpressed in esophageal squamous cell carcinomas, which was positive associated with poor prognosis of esophageal cancer patients. ROC1 knockdown significantly inhibited the growth of esophageal cancer cells *in vitro* and *in vivo*. Mechanistically, ROC1 silencing induced G2 cell cycle arrest and triggered apoptosis by accumulating the pro-apoptotic protein NOXA. Consistently, the downregulation of NOXA expression *via* siRNA substantially attenuated apoptosis induced by ROC1 silencing. These findings suggest that ROC1 is an appealing drug target for esophageal cancer.

## INTRODUCTION

Esophageal cancer (EC) is one of the leading causes of cancer related death in the world and there are about 400,200 people died from EC in 2012 [1]. Esophageal squamous cell carcinoma (ESCC) is a major histological subtype among all types of the esophageal tumors, especially in China [2]. Limitations of the traditional chemotherapeutic treatment and an advanced stage at the time of diagnosis account for the worse prognosis of patients with ESCC [3–5]. It is necessary to identify new anti-ESCC molecular targets and develop novel therapeutic strategies to ameliorate its outcome.

Regulator of Cullins-1 (ROC1), also known as RING box protein-1(RBX1), interacts with different cullin family members, constitutes the catalytic cores and activates CRL/SCF E3 ligases, which regulate different biological process [6, 7]. Recently, ROC1 was reported to be overexpressed in a number of cancers [8–11], which was associated with poor prognosis of several cancers, such as non-muscle-invasive bladder transitional cell carcinoma (NMIBC) [9], gastric cancer [8, 10] and liver cancer [12]. Dysfunction of ROC1 induced embryonic death and abnormal meiosis [13]. These reports suggested that ROC1 could act as an attractive anticancer target. However, there is little known about the expression and role of ROC1 in esophageal cancer.

Here, for the first time, we reported that ROC1 was overexpressed in esophageal squamous cell carcinomas tissues, which is positive correlation with poor prognosis of esophageal cancer patients. Knockdown of ROC1 effectively inhibited cell proliferation *in vitro* and *in vivo*, induced G2 cell cycle arrest and triggered apoptosis by accumulating the p21, p27, WEE1 or pro-apoptotic protein NOXA. These findings revealed the detailed mechanism for proliferation-inhibition effect of ROC1 knockdown and suggested that ROC1 was an appealing drug target for esophageal cancer.

## RESULTS

### ROC1 is overexpressed in ESCC tissues and predicts diminished survival in ESCC patients

**Figure 1:**
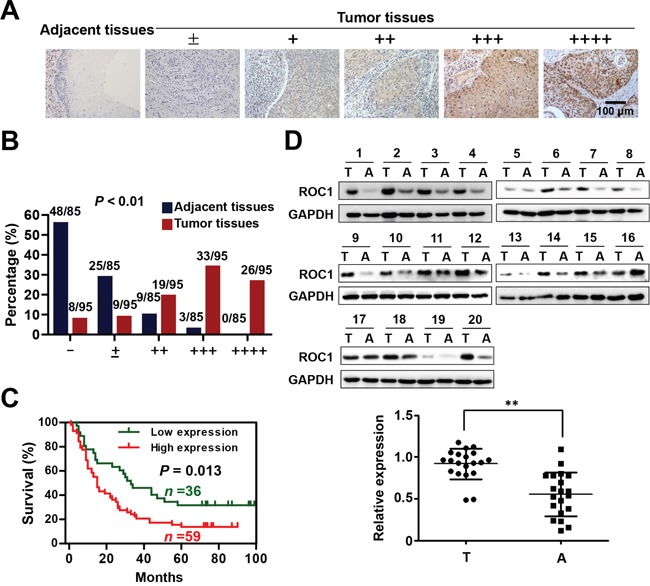
ROC1 is overexpressed in esophageal squamous cell carcinoma tissues **(A)** IHC staining of human esophageal squamous cell carcinoma tissues arrays using ROC1-specific antibodies. According to staining intensity, samples were classified into five groups with increasing staining intensity from the weakest (±, group 1) to the strongest (++++, group 5). **(B)** Classification of tumor samples according to the staining intensity of ROC1. Mann-Whitney Test was used to evaluate the statistical significance of differences between groups. **(C)** Kaplan-Meier curves for the overall survival rate of patients with esophageal squamous cell carcinoma according to the expression of ROC1 (*P* = 0.013, log-rank test). Groups 1-3 was designated as low expression and Groups 4-5 was designated as high expression. **(D)** Western blotting analysis to determine the expression of ROC1 in ESCC tissues and adjacent esophageal tissues. Western blotting results were shown (top panel). Protein expression was quantified and statically analyzed (bottom panel). (Error bar = S.D.). A=adjacent normal tissues; T=tumor tissues.

To investigate the clinical significance of ROC1 in esophageal cancer, we firstly evaluated the expression levels of ROC1 by immunohistochemistry (IHC) staining of the human ESCC tissue arrays. According to the staining intensity, we classified the samples into five groups with increasing staining intensity from the weakest (±, group 1) to the strongest (++++, group 5) (Figure 1A). Statistical analysis demonstrated that ROC1 was overexpressed in ESCC tissues compared with their corresponding adjacent normal tissues (Figure 1B), which was confirmed by Western blotting analysis (Figure 1D). Furthermore, the overexpression of ROC1 protein was negatively correlated with 5-year overall survival rate of ESCC patients determined by Kaplan-Meier analysis (*P* = 0.013, log-rank test) (Figure 1C).

### Knockdown of ROC1 inhibits the proliferation of esophageal cancer cells

**Figure 2:**
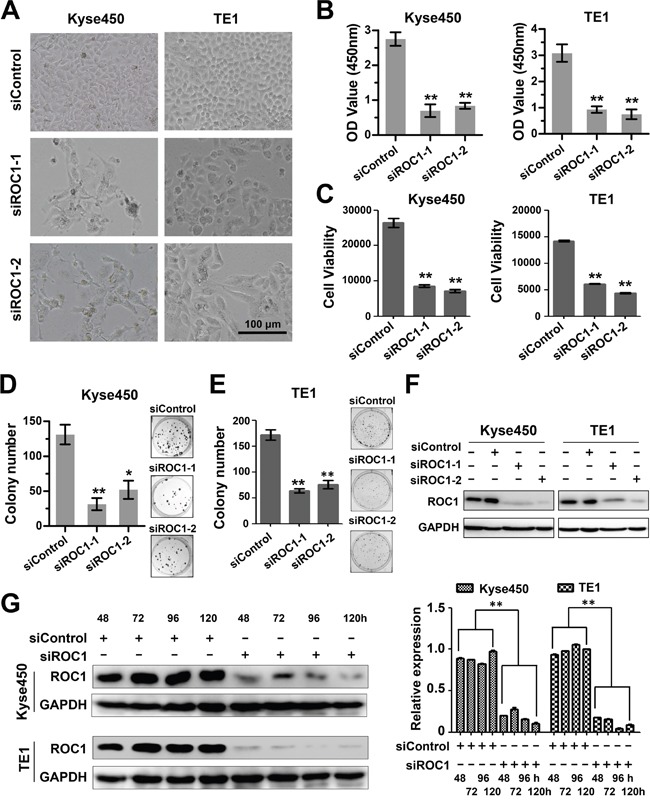
Knockdown of ROC1 inhibited proliferation of human esophageal cancer cells **(A)** Morphology of esophageal cancer cells after silencing ROC1. **(B-C)** Effect of silencing ROC1 on the viability of esophageal squamous cell carcinoma cells TE1 and Kyse450. Cells were transfected with siRNA for 120 h and viability was assessed with the MTT **(B)** and ATPLite **(C)** assay. **(D-E)** The effect of silencing ROC1 on the clonogenic survival of esophageal squamous cell carcinoma cells Kyse450 **(D)** and TE1 **(E)**. Cells were transfected with siRNA for 9 days, and then fixed, stained and counted as described in the Materials and Methods. **(F)** Knockdown efficiency of different siRNAs targeting ROC1. Cells were transfected with siRNA for 96 h and proteins were collected and knockdown efficiency was determined by western blotting. **(G)** Knockdown efficiency of siROC1 at different time points. Cells were transfected with of mixture of siROC1-1 and siROC1-2 for 48, 72, 96 and 120 h and proteins were collected and knockdown efficiency was determined by western blotting (left panel). Protein expression was quantified and statically analyzed (right panel). (Error bar = S.D.).

To further assess the role of ROC1 on cell proliferation of esophageal cancer, ROC1 was knockdown by two specific siRNA oligoes, named siROC1-1 and siROC1-2. Results showed that ROC1 silencing significantly inhibited cell proliferation of both Kyse450 and TE1 cells (Figure 2A-2C). Besides, knockdown of ROC1 also effectively inhibited the cell colony formation in both cell lines (Figure 2D and 2E). The knockdown efficiency of siRNA targeting ROC1 was confirmed by Western blotting assay (Figure 2F and 2G).

### Knockdown of ROC1 induces G2 cell cycle arrest in esophageal cancer cells

**Figure 3:**
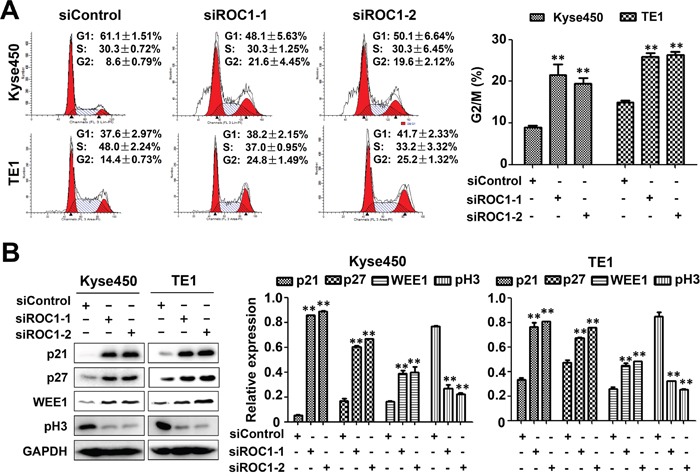
Knockdown of ROC1 induced G2/M cell cycle arrest of human esophageal cancer cells **(A)** TE1 and Kyse450 cells were transfected with siRNA for 48 h, and then stained by PI staining. Cell cycle profile was analyzed by fluorescence-activated cell sorting (FACS) analysis. Representative images were shown (left panel). The statistical significance of differences between groups was assessed using the GraphPad Prism5 software (**P<0.01) were applied (right panel). **(B)** Analysis of cell cycle-related proteins. Cells proteins were collected and cell cycle-related proteins were determined by western blotting (left panel). Protein expression was quantified and statically analyzed (right panel). (Error bar = S.D.).

To elucidate the mechanism of ROC1 knockdown for cell growth inhibition, we firstly examined the cell cycle profile of the ROC1-silencing cells. As shown in Figure 3A, knockdown of ROC1 triggered G2/M cell cycle arrest in both Kyse450 and TE1 cells. Furthermore, ROC1 silencing induced significant accumulation of WEE1, an inhibitor of G2-M phase transition [14], while a decrease of p-H3, a hallmark of M phase cells [15], indicating that ROC1-silencing cells were arrested at the G2 phase (Figure 3B).

### Knockdown of ROC1 triggers apoptosis in esophageal cancer cells

**Figure 4:**
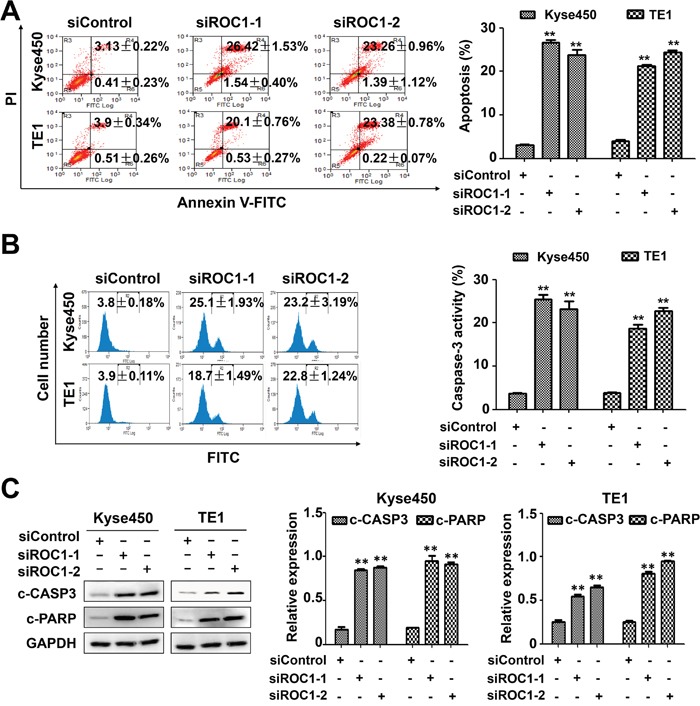
Knockdown of ROC1 triggered apoptosis of human esophageal cancer cells TE1 and Kyse450 cells were transfected with siRNA for 96 h. **(A)** Apoptosis was determined by Annexin V -FITC/PI double-staining analysis. The early apoptotic (Annexin V-FITC positive) and necrotic/late apoptotic (Annexin V-FITC positive, PI positive) were quantified as apoptotic cells. Representative images were shown (left panel). The statistical significance of differences between groups was assessed using the GraphPad Prism5 software (**P<0.01) were applied (right panel). **(B)** Caspase-3 activity was analyzed by FACS. Representative images were shown (left panel). The statistical significance of differences between groups was assessed using the GraphPad Prism5 software (**P<0.01) were applied (right panel). **(C)** Cleaved PARP and caspase-3 were detected by western blotting analysis (left panel). Protein expression was quantified and statically analyzed (Error bar = S.D.) (right panel).

**Figure 5:**
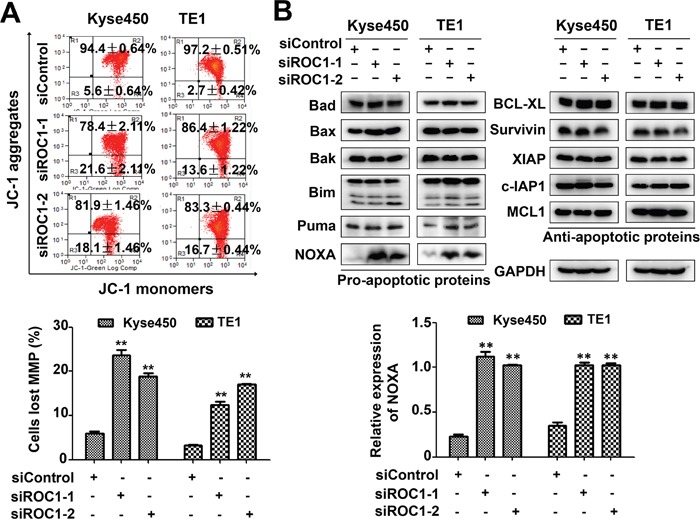
Knockdown of ROC1 induced mitochondrial apoptosis **(A)** Knockdown of ROC1 led to mitochondrial membrane depolarization. Mitochondrial membrane depolarization was detected with mitochondrial membrane potential assay kit with JC-1, according to the manufacturer's protocol. Representative images were shown (top panel). The statistical significance of differences between groups was assessed using the GraphPad Prism5 software (**P<0.01) were applied (bottom panel). MMP stands for mitochondrial membrane potential. **(B)** Effects of ROC1 silencing on the expression of pro-apoptotic and anti-apoptotic proteins. TE1 and Kyse450 cells were transfected with siRNA for 96 h and subjected to western blotting analysis using antibodies against pro-apoptotic and anti-apoptotic proteins. GAPDH served as a loading control (top panel). The expression of NOXA was quantified and statically analyzed (bottom panel).

Next we examined whether apoptosis was also responsible for the anti-proliferative effects of ROC1 silencing. Results showed that knockdown of ROC1 led to a significant increase in Annexin V-positive cells (Figure 4A) and caspase-3-actived cells (Figure 4B). Similarly, ROC1 silencing significantly induced the cleavage of PARP and caspase-3 (Figure 4C). Furthermore, we found that knockdown of ROC1 led to the loss of mitochondrial membrane potential (ΔΨm) (Figure 5A), a classical marker of the activation of intrinsic apoptosis, which suggested that knockdown of ROC1 triggered mitochondrial apoptosis.

### NOXA plays a critical role for ROC1 silencing-induced apoptosis

To explore the potential mechanism of apoptosis, we investigated systematically the effect of ROC1 knockdown on the expression of the pro-apoptotic and anti-apoptotic proteins. Among these proteins, pro-apoptotic protein NOXA was significantly up-regulated in both cell lines (Figure 5B), suggesting that NOXA may be a critical mediator for ROC1-silencing-mediated apoptosis.

**Figure 6:**
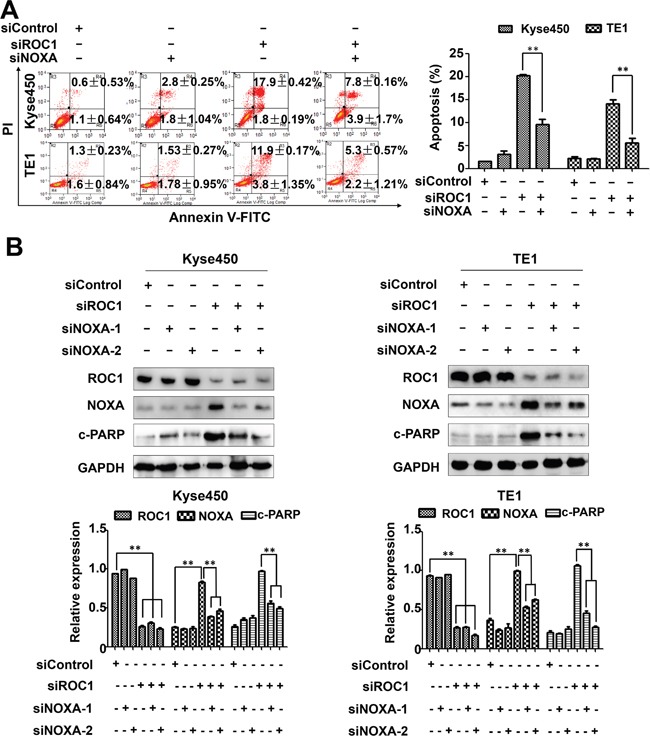
NOXA play an important role in ROC1-silencing induced apoptosis Down-regulation of NOXA reduced ROC1-silencing-induced apoptosis. Kyse450 and TE1 cells were transfected with control siRNA, NOXA siRNA, ROC1 siRNA or both. **(A)** Apoptosis induction was quantified by AnnexinV-FITC/PI double-staining analysis. Representative images were shown (left panel). The statistical significance of differences between groups was assessed using the GraphPad Prism5 software (**P<0.01) were applied (right panel). **(B)** Knockdown efficiency and cleaved PARP were assessed by western blotting analysis (top panel). Protein expression was quantified and statically analyzed (Error bar = S.D.) (bottom panel).

To further define the role of NOXA in ROC1-silencing-induced apoptosis, the expression of NOXA was down-regulated by siRNA in ROC1-silencing cells. NOXA knockdown significantly reduced the induction of apoptosis (Figure 6A) and the cleavage of PARP in ROC1-silencing cells (Figure 6B). These findings highlighted a pivotal role of NOXA in ROC1-silencing-induced apoptosis.

### ROC1 silencing suppresses the growth of human ESCC tumors in murine model

**Figure 7:**
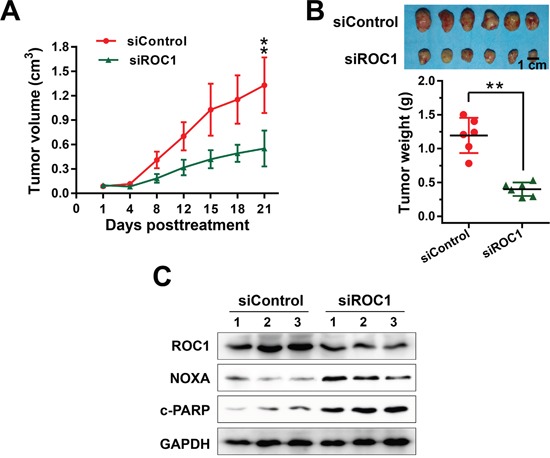
ROC1 silencing suppressed esophageal tumor growth *in vivo* **(A)** Tumor size was determined by caliper measurement. The tumor volume was calculated using the ellipsoid volume formula (Length×Width^2^/2). **(B)** Mice were sacrificed and tumor tissues were harvested, photographed (top panel), and weighed (bottom panel) at the end of study (**, *P* < 0.01. Error bar = S.D.). **(C)** Proteins extracted from tumor tissues were analyzed by western blotting using anti-cleaved PARP, NOXA and ROC1 antibodies. GAPDH was used as a loading control.

After demonstrating the inhibition efficacy of ROC1 silencing *in vitro*, we further investigated the growth-suppressive effect of ROC1 knockdown in subcutaneous-transplantation tumor model of human esophageal cancer in mice. Results showed that ROC1 silencing significantly suppressed tumor growth over time while control tumors grew rapidly, as revealed by the tumor growth curve (Figure 7A, **P<0.01), tumor size (Figure 7B, top panel) and tumor weight analysis (Figure 7B, bottom panel, **P<0.01). Moreover, we further examined whether NOXA and apoptosis were activated *in vivo*. As shown in Figure 7C, ROC1 was effectively downregulated and the expression of NOXA and cleaved PARP were significantly induced upon ROC1 silencing. These observations indicated that ROC1 silencing inhibited esophageal tumor growth both *in vitro* and *in vivo* by accumulating the expression of NOXA.

### ROC1 knockdown enhances the cytotoxity of cisplatin (CDDP) to ESCC cells

**Figure 8:**
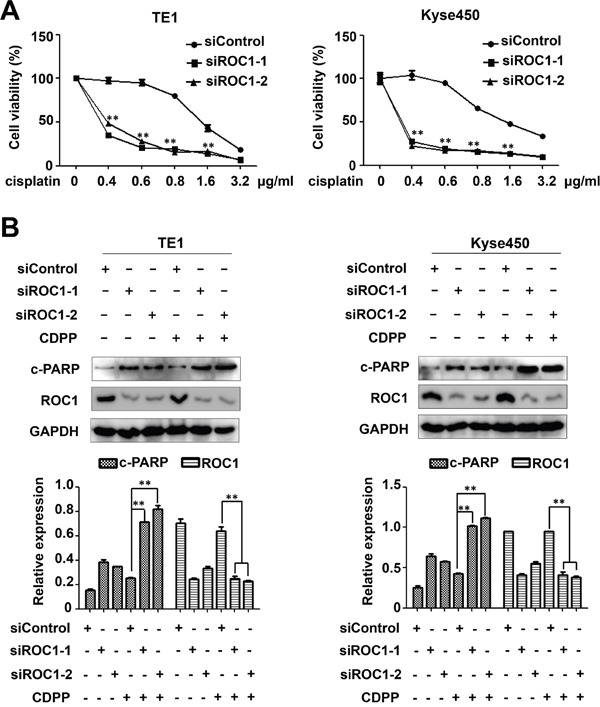
ROC1 silencing enhanced the cytotoxity of cisplatin (CDDP) **(A)** TE1 and Kyse450 cells were transfected with siROC1 for 48 h and then treated with different concentration of CDDP for 48 h. Cell viability was measured using the ATPLite assay. All the data was normalized (**P<0.01; n = 3. Error bar = S.D.). **(B)** Knockdown of ROC1 significantly enhanced CDDP-induced apoptosis. TE1 and Kyse450 cells were transfected with siROC1 for 48 h and then treated with 1μg/ml CDDP for 48 h. Knockdown efficiency and cleaved PARP were assessed by western blotting analysis (top panel). Protein expression was quantified and statically analyzed (Error bar = S.D.) (bottom panel).

To investigate whether targeting ROC1 could act as a novel chemosensitizer to increase the anti-ESCC activity of CDDP, we treated ESCC cells TE1 and Kyse450 with DMSO or CDDP after siROC1 transfection. Results showed that ROC1 silencing significantly enhanced the cytotoxity of CDDP and inhibited cell viability (Figure 8A). Moreover, knockdown of ROC1 significantly enhanced CDDP-induced apoptosis, which is evident by the increased expression of cleaved-PARP (Figure 8B).

## DISCUSSION

Despite the improvement in the therapy for ESCC, the prognosis remains poor and the 5-year survival rate remains lower than 25% [16]. Diagnosis at advanced stage and resistance to chemotherapy still persecute the refractory disease [4]. Therefore, it is urgent to find new therapeutic target.

Recently, some reports showed that ROC1 is overexpressed in multiple human tumors and contributed to the tumor progression [8–12]. RBX1/ROC1 disruption results in early embryonic lethality [13, 17], cancer cell death [11, 17] or the inhibition of tumor cell migration [18]. In the present study, we revealed that ROC1 was hyperexpressed in esophageal squamous cell carcinoma tissues compared to adjacent tissues, and the overexpression of ROC1 was negatively associated with 5-year survival rate of the patients, indicating that ROC1 may play an important role in tumor progression of ESCC. Moreover, knockdown of ROC1 significantly inhibited cell proliferation i*n vitro* and *in vivo*, and enhanced the cytotoxity of CDDP to ESCC cells. These results indicate that ROC1 serves as an attractive anti-ESCC target.

Previous reports showed that knockdown of ROC1 destroy CRL/SCF complexes and thus disrupt CRL/SCF E3 ligase activity. As a result, a trail of CRL substrate were accumulated and thus triggered DNA damage response [19], lead to G2-M cell cycle arrest [11, 19], senescence [11, 12], apoptosis [11] or protective autophagy [12] in a cancer cell-specific manner. In terms of apoptosis induction, Jia's finding showed that proapoptotic protein PUMA are involved in RBX1 (ROC1) silencing-induced apoptosis [11], whereas NOXA is associated with RBX2 silencing [20]. Here we found that knockdown of ROC1 led to the accumulation of NOXA and NOXA knockdown afforded significant protection against apoptosis, implying that NOXA also play an important role in ROC1 silencing-induced apoptosis for ESCC cells.

NOXA has been proven as a particularly relevant therapeutic target for several tumors, such as chronic lymphocytic leukemia (CLL) [21, 22], liver cancer [23], lung cancer [24] and ESCC [25]. As a member of the BH3-only proteins, NOXA triggered apoptosis at mitochondria or the endoplasmic reticulum [26], which has an important role for apoptosis induction by cytotoxic drugs, including ubiquitin proteasome system (UPS) inhibitors [27–32]. Administration of bortezomib significantly increases the expression of NOXA in human melanoma cell grown and inhibits tumor growth [29, 31]. NOXA is an important mediator of bortezomib-induced apoptosis in chronic lymphocytic leukemia cells [27]. Treatment ESCC or acute myelogenous leukemia cells with MLN4924, a neddylation inhibitor, lead to inactivation of CRL/SCF E3 ubiquitin ligase and transactivation of NOXA in a cell-type-specific manner to induce cell apoptosis [25, 33]. These studies suggest that NOXA perhaps play an important role in ROC1 silencing-induced apoptosis in esophageal cancer cells.

In summary, our study provides the first piece of appealing evidence supporting the notion that ROC1 is an attractive target for ESCC cancer as well as serves as a potential prognosis marker.

## MATERIALS AND METHODS

### Cell culture

Human esophageal squamous cell carcinoma cell lines Kyse450 and TE1 were cultured in Dulbecco's Modified Eagle's Medium (Hyclone) containing 10% FBS (Biochrom AG) at 37°C with 5% CO_2_. Cisplatin was purchased from MedChem Express and dissolved in DMSO.

### Immunohistochemistry (IHC) staining of human esophageal cancer tissue array

Human esophageal cancer tissue array was purchased from Shanghai Outdo Biotech Co. Ltd. The detailed clinicopathological characteristics of esophageal squamous cell carcinoma patients are listed in Table 1 for statistical analysis. IHC staining was done with specific ROC1 antibody (Abcam Trading Company Ltd, Shanghai, China) according to our previous reports [24, 25]. Briefly, the tissue array sections (4 μm) were dehydrated and subjected to peroxidase blocking. Primary antibodies were added and incubated at 4°C overnight, followed by staining with a GTVisionTMIII Detection System/Mo&Rb (Gene Tech Company Limited, Shanghai, China). The slides were counterstained with hematoxylin. The stained slides were observed under microscopy, and images were acquired. Based on staining intensity, samples were classified into five groups from the lowest density (±, group 1) to the highest (++++, group 5) [24, 25]. Overall survival was calculated using Kaplan-Meier analysis and compared with the log-rank test. Groups 1-3 designated low expression and Groups 4-5 designated high expression.

**Table 1:** Clinicopathologic parameters according to the expression of ROC1

Variable	Overall No.	ROC1		*P*
		Low	High	
Age (n*=95)				0.832
<60	30	5	25	
≥60	65	12	53	
Gender(n*=95)				0.866
male	73	28	45	
female	22	8	14	
pT (n*=91)				0.698
T1	5	2	3	
T2	12	4	8	
T3	71	24	47	
T4	3	2	1	
pN (n*=94)				0.338
N_0_	45	19	26	
N_1_	49	16	33	
pTMN (n*=91)				0.799
I	6	3	3	
II	41	16	25	
III	38	12	26	
IV	6	2	4	
Histologic grade (n*=95)				0.343
1	30	14	16	
2	43	13	30	
3	22	9	13	

### Collection of esophageal cancer tissues

Fresh primary esophageal cancer tissues and adjacent esophageal tissues were collected from 20 esophageal squamous cell carcinoma patients undergoing resection at the Linzhou Cancer Hospital (Linzhou, Henan, China) from July 2012 to September 2014. The detailed information was showed in Supplementary Table 1. Histological diagnosis and tumor-node-metastasis (TNM) stages of cancers were determined in accordance with the American Joint Committee on Cancer (AJCC) manual criteria for esophageal cancer.

### Gene silencing using small interfering RNA (siRNA)

Kyse450 and TE1 cells were transfected with siRNA oligonucleotides, synthesized by RIBOBIO (Guangzhou, China) using Lipofectamine 2000. The sequences of the siRNA are as follows: siROC1-1: GACUUUCCCUGCUGUUACCUAA [12]; siROC1-2: CUGUGCCAUCUGCAGGAACCACA [12]; siNOXA-1: GUAAUUAUUGACACAUUUC [34]; siNOXA-2: GGUGCACGUUUCAUCAAUUUG [35]; siControl: UUCUCCGAACGUGUCACGU.

### Cell viability and clonogenic survival assay

Kyse450 and TE1 cells were transfected with siControl or siROC1 for 24 h and then seeded in 96-well plates (2.5×10^3^ cells per well) for 120 h. Cell viability was determined by MTT and ATPLite assay according to the manufacturer's protocol.

For the clonogenic assay, cells transfected with siControl or siROC1 were seeded into 6-well plates with 500 cells per well in triplicate and incubated for 9 days. The colonies formed were fixed, stained, and counted. The colonies with more than 50 cells were counted.

### Western blotting

Kyse450 and TE1 cells were transfected with siControl or siROC1 for 96 h and then proteins were collected for western blot analysis, using antibodies against p21, p27, WEE1, pH3, cleaved caspase-3, cleaved poly (ADP) ribose polymerase (PARP), Pro-Apoptosis Bcl-2 Family Antibody Sampler Kit, Pro-Survival Bcl-2 Family Antibody Sampler Kit, IAP Family Antibody Sampler Kit and glyceraldehyde-3-phosphate dehydrogenase (GAPDH) (Cell Signaling, Boston, MA). Densitometric analysis for the quantification relative to GAPDH was performed using the Image J software.

### Cell cycle analysis

Kyse450 and TE1 cells were transfected with siControl or siROC1 for 48 h and then cells were harvested, fixed in 70% ethanol at −20°C, stained with propidium iodide (PI, 50μg/ml, Sigma) containing RNase A (30μg/ml, Sigma) at 37°C for 30 min, and analyzed for cell-cycle profile by flow cytometry (Becton Dickinson FACScan). Data were analyzed with ModFit LT software (Verity).

### Apoptosis assay

Kyse450 and TE1 cells were transfected with siControl or siROC1 for 96 h and then apoptosis was determined with Annexin V-FITC/PI Apoptosis Kit (BioVision, Inc. Milpitas, California) as per the manufacturer's instructions. The early apoptotic (Annexin V-FITC positive) and necrotic/late apoptotic (Annexin V-FITC positive, PI positive) were quantified as apoptotic cells. The activities of caspase-3 were quantitated by CaspGLOW Fluorescein Active Caspase-3 Staining Kit (BioVision, Inc. Milpitas, California) according to the manufacturer's instructions.

### Evaluation of mitochondrial membrane depolarization

Kyse450 and TE1 cells were transfected with siControl or siROC1 and then Mitochondrial Membrane Depolarization was detected with the mitochondrial membrane potential (MMP) assay kit with JC-1 according to the manufacturer's protocol (YeasenInc, Shanghai, China). The data were acquired and analyzed by flow cytometer as described previously [23].

### Tumor formation assay

Tumor formation assay was done according to previous reports [12]. Tumor size was determined by caliper measurement. The tumor volume was calculated using the ellipsoid volume formula (Length×Width^2^/2). Tumor tissues were harvested, photographed, and weighed. Protein expression of tumor tissues was evaluated by western blotting analysis using specific antibodies as indicated. Animal experiments were performed in accordance with the National Institutes of Health Guide for the Care and Use of Laboratory Animals.

### Statistical analysis

The statistical significance of differences between groups was assessed using the GraphPad Prism5 software. The unpaired 2-tailed *t* test was used for the comparison of parameters between groups. The Mann-Whitney test was used for data that are not of normal distribution by SPSS software. The standard deviation (S.D.) value was calculated by Excel software. For all tests, two levels of significance (*P<0.05, **P<0.01) were applied.

## SUPPLEMENTARY MATERIALS TABLES



## Abbreviations

ROC1: regulator of Cullins-1
EC: Esophageal cancer
ESCC: Esophageal squamous cell carcinoma
RBX1: RING box protein-1
IHC: immunohistochemistry
CDDP: cisplatin
UPS: ubiquitin proteasome system
TNM: tumor-node-metastasis
MMP: mitochondrial membrane potential
S.D.: standard deviation
FACS: fluorescence-activated cell sorting
