# Supplementary material for: Targeting the overexpressed ROC1 induces G2 cell cycle arrest and apoptosis in esophageal cancer cells
Source: Oncotarget. 2017 Mar 16;8(17):29125–37. doi: 10.18632/oncotarget.16250 (PMC5438718; doi:10.18632/oncotarget.16250)
Supplement: Supplementary file 1 [file oncotarget-08-29125-s001.pdf]

Targeting the overexpressed ROC1 induces G2 cell cycle arrest and apoptosis in esophageal cancer cells

SUPPLEMENTARY MATERIALS

SUPPLEMENTARY TABLE

Supplementary Table 1: Clinicopathologic parameters of the samples used in the western blotting assay

No.	Gender	Age	TNM	Histologic grade
1	M	48	T1N0M0	2
2	M	62	T1N0M0	2
3	M	55	T2N0M0	2
4	F	72	T1N1M1	1-2
5	M	62	T1N0M0	2
6	F	62	T1N0M0	2-3
7	M	58	T1N0M0	1
8	F	63	T2N0M0	2
9	M	66	T1N2M1	2
10	F	55	T2N1M1	2
11	M	73	T2N2M1	2
12	M	61	T1N1M1	1
13	M	72	T2N0M0	2
14	F	70	T1N1M1	3
15	M	64	T1N0M0	1
16	M	59	T1N0M0	2
17	M	71	T1N0M0	2
18	M	69	T1N0M0	2
19	M	63	T1N2M1	2-3
20	M	60	T2N1M1	2
